# FLOWER: An Approach for Enhancing E-Learning Experience Amid COVID-19

**DOI:** 10.3390/ijerph19073823

**Published:** 2022-03-23

**Authors:** Ibrahim A. Elshaer, Abu Elnasr E. Sobaih

**Affiliations:** 1 Management Department, College of Business Administration, King Faisal University, Al-Ahsaa 31982, Saudi Arabia; 2 Faculty of Tourism and Hotels, Suez Canal University, Ismailia 41522, Egypt; 3 Faculty of Tourism and Hotel Management, Helwan University, Cairo 12612, Egypt

**Keywords:** e-learning, distance learning, positive e-learning experience, COVID-19, medical students, education sustainability, Kingdom of Saudi Arabia

## Abstract

The worldwide COVID-19 pandemic has forced higher institutions to shift towards electronic (e) learning. Despite a plethora of research on the responses of higher education institutions to COVID-19 and their shift towards e-learning, research often focuses on the opportunities and/or challenges of e-learning amid COVID-19. Notwithstanding this, limited research has addressed how e-learning experiences can be enhanced among medical students, who often need conventional leaning, especially for practical courses. This research addresses a gap in the knowledge and examined medical students’ perceptions of e-learning using the Blackboard platform, and the elements or predicators that affect their e-learning experience amid COVID-19 in the Kingdom of Saudi Arabia. An online survey was transmitted to medical students in three main public universities. Based on the findings, a FLOWER model was proposed for improving e-learning experience using Blackboard among medical students. This model includes six dimensions: feedback, leverage to remain motivated, open resources and information, working together, evaluation, and reflection and knowledge. These dimensions are interrelated, and enable the creation of a positive e-learning experience. The results showed that four of the six dimensions have high positive and significant path coefficients: open sources and information; leverage to remain motivated; working together; and reflection and knowledge construction. Two of the six dimensions have low positive, but significant, path coefficients (feedback and evaluation), which require further consideration by policymakers and educators. The results have several theoretical and practical implications, which are elaborated upon.

## 1. Introduction

The World Health Organisation (WHO) declared the novel coronavirus disease 2019 (COVID-19) to be a worldwide pandemic in March 2020. Governments were forced to change traditional face-to-face classrooms to distance learning to control virus outbreaks by maintaining social (place) distancing [1]. Higher education institutions have adopted different technological and digital platforms to ensure quality education similar to that provided in traditional classrooms [2]. Among these technological and digital platforms are the formal online learning management systems (FOLMS), e.g., Blackboard and Moodle, which were designed as learning platforms [3]. Other institutions adopted less costly collaborative digital platforms, e.g., Zoom, Microsoft Teams, and Google Classroom [4], whereas others adopted social networking sites or applications (SNS/A), e.g., Facebook and WhatsApp [5].

The long-lasting impact of the COVID-19 pandemic has forced higher education to continue with either full distance learning or blended learning (a mix of classroom and online learning) [4]. Choosing the proper tool for distance learning is crucial since this requires considering several criteria, such as efficiency, quality of service, cost, and protecting the data of participants, in addition to security [2,6]. Most higher education institutions in the Kingdom of Saudi Arabia have adopted FOLMS amid COVID-19, i.e., Blackboard, to sustain their education. However, despite the fact that FLOMS have helped higher education institutions achieve their first mission—teaching and learning—some issues have arisen about the quality of outcomes compared to traditional classrooms [2,3]. This research is an attempt to answer the following research questions:

1. How did medical students perceive their e-learning using Blackboard amid COVID-19?
2. What are the elements or predicators of the e-learning experience using Blackboard amid COVID-19 for medical students in Saudi Arabia?
3. How can higher education institutions, and their educators, improve e-learning for medical students amid crises, e.g., the COVID-19 pandemic?

The current research explored the perceptions of medical and dental students in public universities in the Kingdom of Saudi Arabia amid COVID-19 regarding their new e-learning experience, and addressed the elements or predicators of the e-learning experience using FOLMS, i.e., Blackboard. The research focuses on understanding the e-learning experience of medical students, who often require conventional learning, especially for practical courses. Assessing and understanding this experience will enable policymakers to enhance the learning process of those students and their learning outcomes. Hence, the structure of this article is as follows. First, it starts with a review and background research about higher education institutions′ responses to COVID-19, especially in relation to teaching and learning. Additionally, it critically reviews previous research regarding students’ learning experiences, especially in relation to distance learning. Second, the article discusses the methods used for data collection and data analysis. Third, it presents the research results. Fourth, it discusses the results and highlights the implications of the research. Fifth, it presents the limitations of the research and highlights the opportunities for further research.

## 2. Literature Review and Hypothesis Building

### 2.1. Higher Education Institutions’ Responses to COVID-19

A plethora of research [1,2,3,4,5,6,7,8,9] has been undertaken to address the higher education institutions’ responses to COVID-19. While some previous research papers have highlighted the opportunities and challenges of distance learning as a response to COVID-19 [1,6,9], other research has highlighted the values of specific digital platforms adopted to ensure proper distance learning amid COVID-19 [2,3,4]. A study on developing countries’ content [1], i.e., India, confirmed the shift to distance learning and the promotion of technology-enabled learning for students, despite the limited resources and poor infrastructure, since this is the sole option for sustaining quality education. The same study confirmed that governments should invest in the digital academic experience. Governments must also adopt a change management process in curricula to reflect changes in knowledge construction and learning experiences during and post COVID-19. This issue was confirmed by other studies, which emphasized that higher education institutions must ensure the curricula is responsive to students’ needs to create positive learning experiences [9]. The value of digital platforms in the transformation undertaken in learning amid COVID-19 cannot be questioned [6,8]. However, the quality of platforms should be considered to achieve learning outcomes and develop students’ skills [3,4,5].

### 2.2. Students’ Perceptions of E-Learning Amid COVID-19

Although some studies [10,11,12,13] found that e-learning did not produce the desired results among higher education students, several other studies [7,8] found that students had a positive experience with e-learning. The study of Adnan and Anwar [10] in the Pakistani context showed that both undergraduate and postgraduate students perceived e-learning negatively due to poor digital service, technical, and monetary issues. These findings were confirmed among high school students in Indonesia, indicating that e-learning cannot produce the desired outcomes in a developing country such as Indonesia. Similarly, in the study of Al-Balas et al. [11], the satisfaction rate among medical students regarding distance learning was poor (26.8%). This percentage was significantly improved among students with previous distance learning experience and live streaming sessions. As a result, the same study concluded that “*understanding technological, financial, institutional, educator, and student barriers is essential for successful distance learning implementation in medical education*” [11], p. 2. Another study by Kaur et al. [13] on medical students in Pakistan showed that, despite students′ reporting some values of e-learning in relation to communication, building skills and knowledge, and better understanding of classes and assignment submission, e-learning is not an effective method for all students compared to face-to-face in-class learning.

By comparison, the study by Anwar et al. [7] on medical and dental students in private colleges in Pakistan showed a positive perception of e-learning among students. Students perceive e-learning as being flexible due to the provision of online study materials and records, and that it saves time. Female students have more positive experiences with e-learning than male students, although both find e-learning stimulates their interests. Hence, students are ready for the shift in education towards e-learning. Another study on nursing students in Nepal [8] showed that they have a positive attitude regarding e-learning despite the technical issues they face with digital learning. The study confirmed that if e-learning becomes user-friendly, by addressing the limited technical issues and intent problems, e-learning may be a great alternative to traditional learning.

### 2.3. Enhancing E-Learaning Experince for Students

Distance learning has been the sole response to COVID-19 and was not an option for higher education institutions [1]. This requires paying sufficient attention to teaching and learning approaches and students’ e-learning experiences [8,9,10,11,12]. Hence, it is crucial to examine how positive e-learning experiences can be enhanced, as was undertaken in this research, especially for medical students. Medical students in developed countries have had positive experiences with e-learning compared to other students in developing counties, where there are challenges with e-learning [14]. The same study [14] found that, although e-learning amid COVID-19 is satisfactory, conventional learning is more appropriate for clinics and laboratories among medial students. Educators agreed that, despite the challenges they face with e-learning during COVID-19 in engaging students and assessing their academic performance, they will integrate e-learning in their future education [15]. Toquero [16] called for innovative strategies to enhance the learning experience among students in these critical times. A review of research on student e-learning experiences [e.g., 11–13] identified several factors that should be considered to ensure a positive e-learning experience for students. These factors are student engagement and working with them, student support and motivation, access to information and resources, assessment and feedback, reading activities and exams, and personal reflection.

Access to information and open resources is important for creating a positive e-learning experience [12]. Interaction and support from educators and peers improve their learning participation [17]. Digital platforms should also encourage students to work together and actively collaborate in learning activities [18]. E-learning often has limitations in relation to evaluation and feedback, especially those that require direct interaction, e.g., in practical medical classes [19]. Hence, this requires paying sufficient attention to feedback in e-learning to create a positive experience. It is also critical to balance theoretical and practical knowledge to ensure a positive e-learning experience for students [13]. Further investigation and examination of these factors, which can create a positive e-learning experience, need to be undertaken, and was the aim of this study.

## 3. Methodology

### 3.1. Sampling

This study’s population was comprised of all medical students enrolled in Saudi Arabian universities. According to Statista, over 20,000 students were enrolled in 20 universities in 2019. This study examined public universities in the Kingdom of Saudi Arabia that extensively rely on the Blackboard platform to give lectures and communicate with students amid the COVID-19 outbreak. To ensure that institutions were neither over- nor under-represented, 500 questionnaires were delivered to each university (King Faisal University in the Eastern region, Imam Mohammad Ibn Saud Islamic University in the Riyadh region, and Umm Al-Qura University in the holy city of Mecca). As a result, an online survey was used to send out 1500 questionnaires. A total of 1200 questionnaires with relevant data for analysis were received, with an overall response rate of roughly 80%.

The research team delivered the questionnaire to students through their personal networks, i.e., educators working in the medical colleges at these universities. They were asked to send the questionnaire’s link to their students through WhatsApp groups or emails. There was no power over students because they were told that the study was only for research purposes and that their answers would be kept anonymous.

Participation was optional and anonymous, and all the required precautions were in place to ensure the confidentiality of the data. To guarantee that respondents could not be recognized, all personally identifiable information about them was removed from the publicly available analysis. Additionally, sensitive items such as their name, age, and the names of their universities were optional.

### 3.2. Development of Instruments and Questionnaires

The questionnaires measured the research dimensions using a multi-item scale (5-point Likert scale). The scale consisted of six formative dimensions derived from Awidi et al. [20]. The scale has 26 items and was designed to describe the six determinants of students’ e-learning experience (FLOWER). This scale hypothesized that the student e-learning experience is improved when students: (**F**) have proper Feedback which aids them to progress their learning; (**L**) have the proper Leverage to remain motivated; (**O**) have **O**pen resources and information; (**W**) **Work** together and collaborate with others; (**E**) have adequate evaluation; and (**R**) have active **R**eflection and knowledge structure for their learning.

The online survey was conducted using the approach described in the literature [21]. Once the instrument was constructed, one member of the research team began constructing the online survey, which was then thoroughly verified for presentation and correctness by other team members prior to delivering the URL to participants. An introduction was produced to define the research’s goal and welcome students to participate. Participants were advised of their confidentiality and the study’s objective. The introduction with the URL (in English and Arabic) was distributed via personal emails and/or different social media accounts to students during November 2021 and lasted for four weeks. Daily, the research team checked and followed up on the replies. At the conclusion of the introduction, contact information (i.e., name, telephone number, email address, and social media profiles) was included for any additional inquiries.

Following the questionnaire’s translation from English (the original language) to Arabic (the respondents’ native language), ten students and ten academics were invited to review the questions for clarity, simplicity, appropriateness, and necessity. During this process, no substantial modifications were made, but a few suggestions for increasing the clarity of the text were included. Cronbach’s alpha (α) scores were used to determine the reliability of the scale items. These values varied from 0.917 to 0.969, above cut-off value of 0.7 proposed by Nunnally [22].

Due to the fact that the data were collected via a self-reporting questionnaire, many procedures were taken to address and examine the possible issue of common method variance (CMV) [23]. Numerous steps were used to reduce CMV during the questionnaire design phase [24]. For instance, the dependent variables preceded the independent factors [21], and respondents′ identity and confidentiality were ensured. Harman′s single factor test was also performed, with all indicators using SPSS software for exploratory factor analysis (EFA), and the number of retrieved factors limited to one without employing the rotation approach. Only one dimension retrieved explained 36.5 percent of the variance, indicating that this component did not account for a significant portion of the variation; hence, CMV was not a concern in this study [25].

The large sample size of 1200 participants was sufficient for analyzing data using structural equation modeling (SEM) [26]. The primary benefit of such a high number is that it enables the use of advanced data analysis techniques such as SEM. This permitted a satisfactory investigation of the interdependent assumptions underlying the research variables in this study.

### 3.3. Data Analysis Techniques

This study employed a variety of data analysis techniques, including preliminary analysis (missing data, normality testing, and sample size estimation); descriptive analysis (respondent characteristics, mean, and standard deviation); and multivariate analysis, which included confirmatory factor analysis (CFA) and SEM. SEM was chosen as the primary data analysis approach due to its unique capacity to integrate factor analysis and linear regression in order to analyze and concurrently test the study′s complex interrelationships between latent/unobserved multidimensional components. Additionally, SEM may be used to analyze links between study variables while taking into account the measurement error associated with poor variable measurement [27]. To determine the SEM goodness of fit (GOF), the following cut-off points were used: 2/df, SRMR (“Standard Root Mean Residual”), RMSEA (“Root Mean Square Error of Approximation”), CFI (“comparative fit index”), NFI (“Normed Fit Index”), TLI (“Tucker–Lewis Index”), PCFI (“Parsimony Comparative Fit Index”), and PNFI (“Parsimony Normed Fit Index”), as recommended by several scholars [26,28,29]. SPSS version 25 and AMOS version 18 software were used throughout the data analysis procedure.

## 4. Results

### 4.1. Preliminary and Descriptive Analysis

SPSS version 25 was used to identify the lowest and maximum values in order to deal with missing data. There were just a few missing data points discovered (less than 5 percent). As a result, the issue of missing data was unimportant, and any technique for dealing with it would give comparable findings [30]. The skewness and kurtosis (distribution of scores) readings indicated that there were no values greater than −2 or +2, indicating a normal univariate distribution [29].

With regard to the profile of respondents, the share of male respondents (47%) was almost equal to that of female (53%) students in this study, with a somewhat greater number of men (65%) in the early years (students in years one and two). As might be expected, the majority of students were under the age of 25 (95 percent). All students were from colleges of medicine.

The participants’ replies ranged from 5 to 1, with 5 indicating “strongly agree” and 1 indicating “strongly disagree”. The mean values varied from 3.80 to 4.15, and the standard deviation values ranged from 0.782 to 1.107, indicating that the data was more dispersed and less concentrated around the mean [31]. Furthermore, as shown in Table 1, variance inflation factor (VIF) values for all variables were less than 0.4, which indicates that multicollinearity was not a problem in this study [26].

### 4.2. Multivariate Data Analysis

A first-order confirmatory factor analysis (CFA) was conducted using the Maximum Likelihood Estimation Method (ML) to determine the constructs’ convergent and discriminant validity. The findings of the first-order CFA analysis indicated that the model fit well (see Table 2). As previously stated, construct reliability was determined using Cronbach’s alpha values and composite reliability (CR). The CR of the six determinants of e-learning experience were as follows: feedback (0.977), leverage to remain motivated (0.976), open resources and information (0.966), working together (0.961), evaluation (0.974), and reflection and knowledge structure (0.982). As a result, all were more than the specified cut-off value of 0.70, indicating acceptable internal consistency [23].

Additionally, the results demonstrated the scales’ convergent validity for two reasons. First, all factor loadings were adequately significant and high, ranging from 0.886 to 0.974 (see Table 2), above the threshold of 0.50 suggested by Hair et al. [26]. Second, the retrieved values of the average variance (AVE) for the constructs (feedback, leverage to remain motivated, open resources and information, working together, evaluation, and reflection and knowledge structure) were 0.914, 0.911, 0.875, 0.862, 880, and 0.917, respectively (see Table 2). All the values were above 0.50, indicating strong convergent validity, as proposed by Hair et al. [26]. The maximum shared variance (MSV) values were similarly lower than the AVE values (see Table 2), indicating that the dimensions had a high degree of discriminant validity [26]. As demonstrated in Table 2, the square root values of the AVE values for each dimension were larger than the intercorrelation values between dimensions, further implying a high degree of discriminant validity [28,29].

### 4.3. Structural Equation Modeling

This study used a confirmatory technique, in which a theoretical model was developed based on a literature review and then empirical data were obtained to assess if they fit the previously established conceptual model highlighted in the methodology section [23]. The theoretical (structural) model is rejected or approved in this phase depending on its compliance with a model fit standard. The SEM analysis demonstrated that the structural model well suited the data: “(χ2 (293, *n* = 1200) = 1166.433, *p* < 0.001, normed χ2 = 3.981, RMSEA = 0.037, SRMR = 0.0401, CFI = 0.941, TLI = 0.946, NFI = 0.942, PCFI = 0.801 and PNFI = 0.806)”, as shown in Table 3.

**Figure 1:**
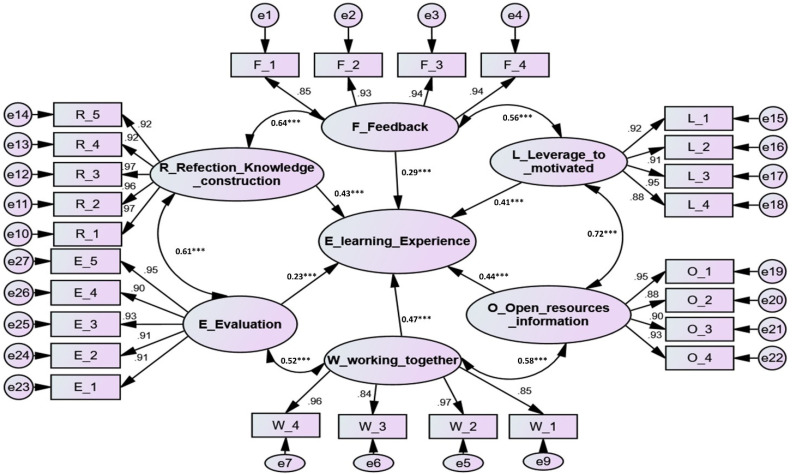
Structural model. Note: ***: significant level less than 0.001.

The research hypotheses were explored after obtaining appropriate criteria for model fit. Each path between latent unobserved variables in the structural model reflects a research hypothesis (see Figure 1). This study provided six path coefficients and six correlations. All of the path coefficients and correlations, with their associated *p*-values, were supported (see Table 3), with details outlined in the following paragraphs.

As shown in Figure 1 and Table 3, the Amos output revealed that two of the six FLOWER dimensions to improve the e-learning experience were found to have low positive but significant path coefficients: feedback (β1 = 0.29, *t*-value = 6.770 with *p* < 0.001); evaluation (β1 = 0.23, *t*-value = 4.522 with *p* < 0.001). However, four of the six FLOWER dimensions to improve the e-learning experience were found to have high positive and significant path coefficients: Leverage to remain motivated (β = 0.41, *t*-value = 8.987 with *p* < 0.001); open resources and information (β = 0.44, *t*-value = 9.753 with *p* < 0.001); working together (β = 0.47, *t*-value = 12.345 with *p* < 0.001); and reflection and knowledge construction (β = 0.43, *t*-value = 9.938 with *p* < 0.001).

Additionally, the findings verified the strong and significant positive correlations between the FLOWER dimensions to improve the e-learning experience: feedback and leverage to remain motivated correlation (r = 0.56, *t*-value = 18.814 with *p* < 0.001); leverage to remain motivated and open resources and information correlation (r = 0.72, *t*-value = 23.305 with *p* < 0.001); open resources and information and working together correlation (r = 0.58, *t*-value = 18.246 with *p* < 0.001); working together and evaluation correlation (r = 0.52, *t*-value = 15.518 with *p* < 0.001); evaluation and reflection and knowledge structure correlation (r = 0.61, *t*-value = 15.977 with *p* < 0.001); and reflection and knowledge structure and feedback correlation (r = 0.64, *t*-value = 19.760 with *p* < 0.001).

Additionally, the FLOWER dimensions in the structural model had a high degree of explanatory power (R2), accounting for 91 percent of the improvement in the e-learning experience (Table 3).

## 5. Discussion and Implications

Based on a sample of public universities in the Kingdom of Saudi Arabia, this research empirically addressed the perceptions of medical students regarding e-learning using the Blackboard platform and the predicators of their e-learning experience amid COVID-19. More specifically, the research examined how the e-learning experience of medical students can be enhanced to support better learning outcomes. The COVID-19 pandemic has forced higher education institutions to shift towards e-learning. However, enhancing e-learning amid COVID-19 has not been properly addressed by scholars to ensure a quality learning process, and to assess implications for future education. Education in medicine requires equipping students with both theoretical knowledge and clinical skills, which makes e-learning more challenging for policymakers, educators, and students. This is mainly due to the limitations of e-learning platforms in the provision of practical courses and clinical sessions, which often require conventional learning with a traditional classroom [13].

The results of the current research showed that four of the six dimensions of the FLOWER model have highly positive and significant path coefficients, namely: open sources and information, leverage to remain motivated, working together, and reflection and knowledge construction. This means that these four dimensions significantly affect their e-learning experience. The findings are consistent with previous research [12,17,18]. It has been argued that the availability of information and resources online is among the dimensions that create a positive e-learning experience [12]. Additionally, for creating a positive e-learning experience, leverage to remain motivated and collaboration with peers enhances the e-learning experience [17,18]. Furthermore, two of the six dimensions of the FLOWER model (feedback and evaluation) have low positive, but significant, path coefficients. Medical students did not find the feedback and evaluation of their work they expected. They require interactive feedback and evaluation similar to those provided in the classroom. Previous research [19] confirmed the limitations of e-learning in relation to the feedback given to medical students, especially in practical courses. This is often because students in practical courses and clinical sessions pay attention to body language and facial expressions when they receive feedback.

Additionally, the study showed a strong and significant positive association between the six dimensions of the FLOWER model, which implies that these dimensions are interrelated. The six dimensions of the FLOWER model—feedback, leverage to remain motivated, open resources and information, working together, evaluation, and reflection and knowledge—together create a positive e-learning experience. This means that a positive e-learning experience can be ensured if these six dimensions are achieved.

The above results have several theoretical implications for scholars in higher education and practical implications for policymakers and educators in higher education. With regard to the theoretical implications, the contradictory results about students’ perceptions of e-learning as positive, inadequate, or negative cannot be generalized to all contexts. This is because the reasons behind these perceptions are also well-known and differ even within the same country [14]. For example, although the study of Kaur et al. [13] on medical students in public institutions showed that e-learning is not an effective method for all students compared to face-to-face learning in class, the study of Anwar et al. [7] on medical and dental students in private colleges showed a positive perception of e-learning among students. These differences in perception were moderated by the infrastructure and online services provided by higher education institutions. Hence, e-learning has been an urgent response to COVID-19, and is not competing with face-to-face classroom instruction because the advantages and disadvantages of both learning approaches are well-documented. It is vital for scholars to understand how these disadvantages can be addressed to create a better e-learning experience for students and achieve proper learning outcomes. As noted by Toquero [16], there is a need for innovative strategies to enhance the e-learning experience of students and support better leaning outcomes. This research is an attempt to understand how positive e-learning can be enhanced, especially for medical students. The research provided six dimensions to be considered by educators for better learning experience among medical students of different genders [32]. Regarding the practical implications, policymakers in higher education should consider adopting blended learning, which includes a mix of online (e-learning) and offline (traditional classrooms) [33,34] to harvest the benefits of both approaches to learning. Educators need to pay better attention to course redesign to fit with e-learning [1] and ensure more engagement with students [18]. Sufficient attention needs to be paid to the evaluation of students’ course work, activities, and exams, in addition to the feedback given to students, because these two dimensions were found to be limited but crucial for medical students, especially in relation to practical courses.

## 6. Limitation and Areas for Further Research

The limitations of this study are likely to be addressed in future research. This study examined the **FLOWER** model (Feedback, Leverage to remain motivated, Open resources, and information, Working together, Evaluation, and Reflection and knowledge) as determinants of the e-learning experience. However, there may be several other factors, such as family support, student self-efficacy, and change resistance, that may also affect the e-learning experience and were not included in the current research. Future researchers are advised to expand the scope of this research in the future by analyzing a wider number of elements affecting the e-learning experience.

Additionally, further research may be conducted to analyze not just the determinants of the e-learning experience, but also the consequences, such as student performance and satisfaction. Furthermore, due to the cross-sectional characteristics of the data, causal correlations between variables could not be inferred precisely. Additionally, although we attempted to avoid the CMV problem in accordance with the recommendations of [35], future researchers may employ longitudinal data or a mix of data sources to validate the study′s suggested model. Finally, by employing a multi-group analysis technique, the suggested model may be utilized for investigations in a variety of contexts (industries or nations) [36].

## Figures and Tables

**Table 1:** Descriptive analysis (*n* = 1200).

Abb.	Items	Min.	Max.	*M*	S. D	VIF	Skewness	Kurtosis
F_ Feedback (Awidi et al. [20])								
F_1	“I was given adequate feedback about how well I was doing in the studied courses.”	1	5	4.08	0.952	3.702	−1.432-	1.281
F_2	“I have been provided with feedback in the studied courses.”	1	5	4.15	0.843	3.738	−1.141-	1.259
F_3	“The feedback on my work gave me direction on how I needed to improve.”	1	5	4.11	0.875	3.882	−1.228-	1.924
F_4	“I used the feedback to improve on the quality of my assignments.”	1	6	4.15	0.867	3.999	−1.339-	1.216
L_ Leverage to remain motivated (Awidi et al. [20])								
L_1	“I am more interested in the studied courses now than when I first started the course.”	1	5	4.07	0.950	3.297	−1.321-	1.925
L_2	“The course structure leverage my ability to successfully achieve the course outcome.”	1	5	4.15	0.782	3.087	–0.883-	1.039
L_3	“The course coordinator was responsive to my learning needs of the course.”			4.09	0.922	3.030	−1.231-	1.898
L_4	“I did feel supported to conduct my own learning through research.”	1	5	4.03	0.936	3.176	−1.200-	1.683
O_Open resources & information (Awidi et al. [20])								
O_1	“I did find the course readings interesting.”	1	5	3.99	0.904	3.178	−0.693-	0.108
O_2	“The online readings really supported my learning.”	1	5	3.95	0.946	3.614	−0.827-	0.547
O_3	“I had access to adequate learning resources relevant for the course.”	1	5	3.96	0.927	3.886	−0.799-	0.543
O_4	“I was provided with sufficient information to get on with my studies.”	1	5	3.97	0.929	3.715	−0.771-	0.388
W_Working together (Awidi et al. [20])								
W_1	“I did find the online working together activities of the course interesting.”	1	5	3.73	0.938	3.421	−0.608-	0.248
W_2	“I felt encouraged by the learning activities provided.”	1	5	3.80	0.891	3.157	−0.836-	0.569
W_3	“I did feel encouraged to learn by engaging in the group activities.”	1	5	3.92	0.939	3.933	−0.752-	0.204
W_4	“I feel a greater sense of community with my class peers.”	1	5	3.80	0.913	3.657	−0.824-	0.410
E_Evaluation (Awidi et al. [20])								
E_1	“The online assignments have enhanced my ability to judge my own work.”	1	5	3.88	1.095	3.900	−0.957-	0.199
E_2	“Assessment in this course improved my learning of the subject.”	1	5	3.88	1.089	3.381	−1.021-	0.414
E_3	“Assessment items were used to improve my learning in this course.”	1	5	3.86	1.107	3.564	−0.926-	0.073
E_4	“The assessment criteria were clearly communicated to me.”	1	5	3.90	1.085	3.085	−1.052-	0.468
E_5	“Preparing for the assessment activities did help my learning of the course goals.”	1	5	3.90	1.068	3.361	−0.998-	0.351
R_Reflection & knowledge structure (Awidi et al. [20])								
R_1	“I feel more confident in articulating and presenting design ideas.”	1	5	4.00	0.986	3.661	−1.330-	1.732
R_2	“I am learning to creatively interpret the legacy of the past through the online design activities.”	1	5	3.99	0.984	2.272	−1.330-	1.740
R_3	“I am gaining insight into how the studied cources engaged with cultural, political and social issues.”	1	5	4.00	0.981	2.685	−1.344-	1.812
R_4	“I felt confident to explore more content of interest of the course.”	1	5	3.95	0.948	2.136	−1.190-	1.620
R_5	“I felt confident in using knowledge acquired from the course to solve problems.”	1	5	3.95	0.944	3.705	−1.186-	1.625

**Table 2:** Discriminant and convergent validity of the measurement model.

Factors and Items	Loading	CR	AVE	MSV	1	2	3	4	5	6
**Feedback (*a* = 0.965)**		0.977	0.914	0.428	**0.956**					
F_1	0.926									
F_2	0.963									
F_3	0.969									
F_4	0.966									
**Leverage to remain motivated** **(*a* = 0.947)**		0.976	0.911	0.428	0.599	**0.954**				
L_1	0.958									
L_2	0.951									
L_3	0.974									
L_4	0.934									
**Open resources & information** **(*a* = 0.972)**		0.966	0.875	0.612	0.599	0.578	**0.935**			
O_1	0.963									
O_2	0.910									
O_3	0.920									
O_4	0.948									
**Working together (*a* = 0.978)**		0.961	0.862	0.406	0.585	0.414		**0.928**		
W_1	0.886									
W_2	0.976									
W_3	0.875									
W_4	0.971									
**Evaluation**		0.974	0.880	0.612	0.578	0.503		0.637	**0.938**	
E_1	0.933									
E_2	0.928									
E_3	0.948									
E_4	0.921									
E_5	0.961									
**Reflection & knowledge structure**		0.982	0.917	0.394	0.513	0.401		0.628	0.501	**0.957**
R_1	0.976									
R_2	0.969									
R_3	0.975									
R_4	0.930									
R_5	0.936									

Model fit: “(χ^2^ (285, *n* = 1200) = 878.085, *p* < 0.001, normed χ^2^ = 3.081, RMSEA = 0.031, SRMR = 0.033, CFI = 0.961, TLI = 0.948, NFI = 0.962, PCFI = 0.801 and PNFI = 0.789)”.

**Table 3:** Results of the hypothesized model.

	Research Model		
	Beta (β)	C-R (T-Value)	SMC
**Path coeffecients**			
Feedback → E-learning experience	0.29 ***	6.770	------
Leverage to remain motivated → E-learning experience	0.41 ***	8.987	------
Open resources and Information → E-learning experience	0.44 ***	9.753	------
Working together → E-learning experience	0.47 ***	12.345	------
Evaluation → E-learning experience	0.23 ***	4.522	-------
Reflection and knowledge construction → E-learning experience	0.43 ***	9.938	------
**Correlations**			
Feedback ⟷ Leverage to remain motivated	0.56 ***	18.814	
Leverage to remain motivated ⟷ Open resources and information	0.72 ***	23.305	
Open resources and Information ⟷ Working together	0.58 ***	18.246	
Working together ⟷ Evaluation	0.52 ***	15.518	
Evaluation ⟷ Reflection and knowledge construction	0.61 ***	15.977	
Reflection and knowledge construction ⟷ Feedback	0.64 ***	19.760	
E-learning experience			0.91

Model fit: “(χ^2^ (293, *n* = 1200) = 1166.433, *p* < 0.001, normed χ^2^ = 3.981, RMSEA = 0.037, SRMR = 0.0401, CFI = 0.941, TLI = 0.946, NFI = 0.942, PCFI = 0.801 and PNFI = 0.806)”. Note: ***: significant level less than 0.001

## Data Availability

Data is available upon request from researchers who meet the eligibility criteria. Kindly contact the first author privately through the e-mail.
